# “A systematic review of mesh support of the breast in aesthetic breast surgery”

**DOI:** 10.1016/j.jpra.2025.06.001

**Published:** 2025-06-04

**Authors:** Grace Ho-Kiu Wong, Stephen Hamilton

**Affiliations:** University College London, London, United Kingdom

**Keywords:** Mammaplasty, Breast implants, Surgical mesh, Aesthetic surgery, Treatment outcome

## Abstract

Aesthetic breast surgeries, including reduction mammoplasty, mastopexy, and breast augmentation, aim to enhance patient satisfaction by improving breast aesthetics. The use of mesh in these surgeries has been proposed to provide improved long-term structural support, addressing conditions such as recurrent ptosis and implant displacement. This narrative systematic review analysed 31 studies involving 2,425 patients to evaluate the effectiveness and safety of mesh in aesthetic breast surgeries. The studies, comprising mostly retrospective case series and a few prospective observational studies, generally reported favourable outcomes with improved breast shape and high patient satisfaction. Reported complication rates were low, with issues such as seroma, haematoma, and infection. However, the evidence is insufficient to recommend the routine use of mesh in aesthetic breast surgery. Future research should focus on high-quality, unbiased studies with standardised outcome measures.

## Introduction

Aesthetic breast surgery aims to enhance patient satisfaction by aligning surgical outcomes with individual aesthetic perceptions. With its growing popularity, the field continues to evolve, incorporating advancements in surgical techniques to achieve more refined and durable results.^1^ These procedures primarily address concerns related to breast shape, volume, and ptosis, catering to patients seeking enhancement, correction, or restoration of their breast aesthetics.

The primary categories of aesthetic breast surgery include breast augmentation, reduction mammoplasty and mastopexy.^2^ Additionally, revisional aesthetic breast procedures are increasingly performed to correct changes in breast shape or volume following primary surgery, ensuring continued patient satisfaction.

Achieving a successful long-term outcome depends on combination of patient-specific factors and surgical techniques. However, the natural aging process and the inherent limitations of breast implants are critical considerations, as they can contribute to progressive changes in breast shape, volume loss, or recurrent loss of projection over time. Understanding these long-term effects is essential for both surgeons and patients in setting realistic expectations and optimising surgical planning to achieve durable aesthetic results.

The use of mesh in implant-based breast reconstruction has gained popularity since 2013, with an increase in the variety of mesh materials licensed for use in these operations.^3^ High-quality systematic reviews and randomised controlled trials have been conducted to evaluate the effectiveness and safety of mesh use in implant-based breast reconstruction.^4^, ^5^, ^6^, ^7^, ^8^ However, evidence supporting the use of mesh in aesthetic breast surgery remains limited. To the best of our knowledge, there is no previous systematic review on the use of mesh in aesthetic breast surgeries.

Meshes have been proposed as a reinforcement to improve the stability of aesthetic breast surgeries, to offer improved long-term structural support and potentially reduce complications such as recurrent ptosis and implant displacement.^9^ Mesh materials available for use in aesthetic breast surgeries can be broadly categorised into synthetic mesh and biologic mesh. Biological meshes are manufactured with animal tissue, while synthetic meshes are composed of absorbable or non-absorbable materials. Despite the potential benefits proposed by mesh manufacturers, there is variability in clinical practice regarding the use of mesh in aesthetic breast surgeries. While an international guideline was issued by a professional body regarding the use of mesh in breast reconstruction procedures,^10^ there is a lack of clinical guidelines regarding the use of mesh in aesthetic breast surgeries.

This narrative systematic review seeks to analyse current evidence on the safety and effectiveness of mesh in aesthetic breast surgeries, addressing key surgical outcomes and complications. By examining the reported outcomes in these studies, this review seeks to provide a comprehensive understanding of the current evidence, identify limitations in current knowledge about mesh use in aesthetic breast surgeries, and suggest directions for future research.

## Aims

This narrative systematic review aims to analyse the available evidence in the literature on the effectiveness and safety of the use of mesh in aesthetic breast surgeries, including mastopexy, breast augmentation and augmentation-mastopexy. We also aim to explore the limitations in current evidence in the published data, and the potential future directions for improving the surgical outcomes in aesthetic breast surgery.

## Materials and methods

### Search strategy

**Figure 1:**
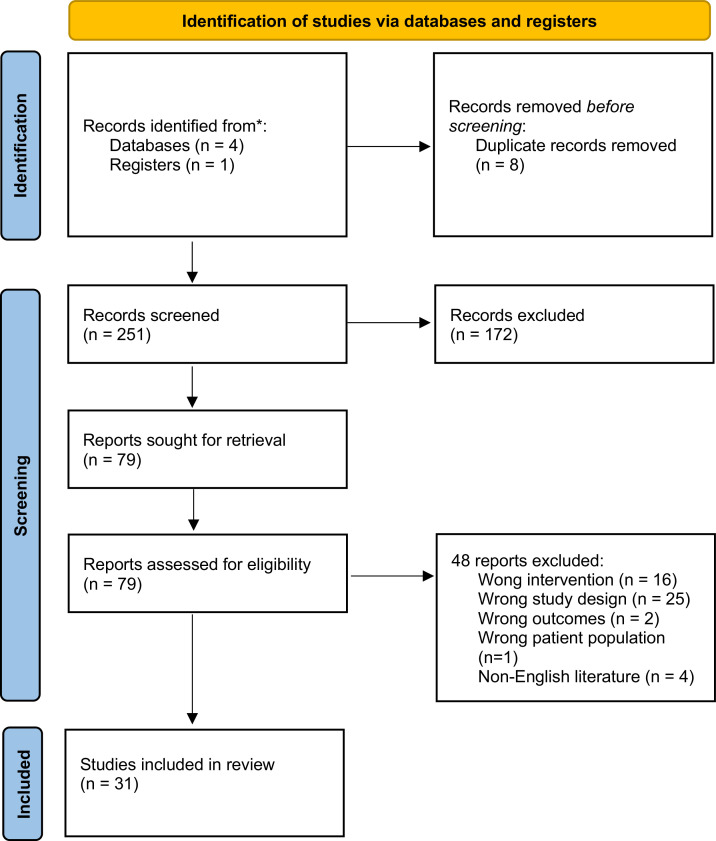
Systematic literature search flow diagram.

This review was performed following a systematic literature search (Figure 1). A broad search of the literature was conducted using the following electronic databases: MEDLINE, EMBASE, Cochrane Central Register of Controlled Trials, and PubMed. The key words used in the literature search were a combination of (mastopexy OR mammoplasty OR breast reduction OR breast augmentation OR aesthetic breast surgery) AND (mesh OR scaffold OR ADM). In addition, the references from relevant studies were reviewed and included if applicable.

### Eligibility

Studies were considered for inclusion if they reported original clinical data on outcomes of aesthetic breast surgeries with use of mesh. Studies published in English language, prior to February 2025 (date of literature search) were included. Review articles, meta-analyses, case reports, editorials, and letters were excluded. Two authors reviewed the articles independently of each other, and the included studies were determined after screening of titles and abstracts, followed by full-text reviews of selected articles.

### Data extraction

Full-text analyses of included studies were performed, with extraction of information summarised in Table 1. The following information was analysed, synthesised, and extracted from each included study: author, year of publication, study design and mean follow-up duration, conflict of interest, surgical technique, mesh support, and surgical outcome. Due to the heterogeneity of data and study design, a meta-analysis could not be conducted. A narrative systematic review was conducted.

**Table 1:** Summary of included studies.

Author	Number of patients	Study design and mean follow-up	Conflict of interest / financial association	Surgical technique	Mesh support	Surgical outcome
Johnson^11^	43	Retrospective review; 2 years	N/A	Central core reduction	Marlex mesh attached to second rib	No complication
Góes^12^	96	Retrospective review; 2–7 years	N/A	Periareolar mammoplasty	-Polyglactine 910 mesh (*n* = 55) -Mixed mesh (polyglactine with polyester) (*n* = 41) -Dermal flap (*n* = 158)	Complications:Seroma: 1.5 % (*n* = 3) -Haematoma: 1 % (*n* = 2) -Fat necrosis cysts: 2 % (*n* = 6) -Loss of areolar sensitivity: 4 % (*n* = 10) Polyglactine mesh: Less drop, less frequent early ptosis, more beautiful mammary shape
deBruijin^13^	170 (327 breasts)	Retrospective review; 1.4 years	Yes	Mastopexy: Lejour / Lejour with small horizontal extension / wise pattern	3D preshaped woven polyester mesh (apside)	No recurrent ptosis
deBruijin^14^	63 (121 breasts)	Retrospective review; 2 years	Yes	Mastopexy	Soft-knitted 3D polyester mesh (Breform)	No major complications or extrusion. 4 patients required minor secondary cosmetic correction.
Maxwell^15^	78	Retrospective review; 12 months	Yes	Revisional breast augmentation; augmentation mastopexy	ADM	−2 complications (2.5 %) requiring reoperation -No Baker III/IV capsular contracture
Góes^16^	5	Prospective observational study; 5 years	N/A	Periareolar mammoplasty	FortaPerm biologic tissue matrix (3-layered)	4 out of 5 (80 %): no asymmetry or ptosis; Bilateral seromas + extrusion of mesh (*n* = 2)
Brown^17^	27	Retrospective review; 19 months	N/A	Wise pattern reduction mammoplasty	ADM mesh sutured to chest wall as a sling or internal brasserie to support the inferior pedicle	Complications:Cellulitis (*n* = 1): resolved with antibiotics -Partial skin flap necrosis (*n* = 1): treated conservatively No significant pseudoptosis or nipple rotation
vanDeventer^18^	112 (218 breasts)	Initial pilot study with 15 patients then retrospective review of 112 patients; 2 years 2 months	Yes	Mastopexy (*n* = 156) Breast reduction (*n* = 62)	Biocompatible mesh, U-shaped mesh anchored to chest wall	Results from pilot study:Loss of nipple sensation (7.6 %) -Fat necrosis (7.6 %) -Infection (3.8 %)
Maxwell^19^	186 (197 breasts)	Retrospective review; 3.3 years	Yes	Revisional breast augmentation or augmentation mastopexy	ADM	98 % revisions reported as successful. Complications: 4.8 %
Pozner^20^	93 (179 breasts)	Retrospective review	Yes	Revisional breast augmentation with or without mastopexy	ADM	Major complications (1.6 %) High-riding implants (*n* = 2) Minor imperfections requiring office procedures (*n* = 7)
AdamsWPJr^21^	11	Prospective observational study; 12 months	Yes	Mastopexy	GalaFLEX (P4HB) mesh	No major complications. Delayed wound healing (*n* = 1). Distance (notch to lowest point of breas): No statistically significant change between months 3 and 12
AdamsWPJr^22^	62	Retrospective review; 12 months	Yes	Mastopexy (*n* = 44) Breast reduction (*n* = 10) Mastopexy + reduction (*n* = 7) Breast augmentation + mastopexy (*n* = 1)	GalaFLEX (P4HB) mesh	89.7 % had successful ptosis correction and maintenance. 5 adverse events (8 %)
Pompei^23^	6	Retrospective review; 12 months	N/A	2 mastopexies and 4 breast reductions	TIGR® Matrix Surgical Mesh	Lower rate of revisional and mesh explantation surgeries.
Ruff^24^	5	Retrospective review; 25 months	N/A	Revisional breast augmentation	TYRX™ absorbable antibiotic-impregnated polypropylene mesh anterior to implant. Triple antibiotic solution of bacitracin (50,000 U)/vancomycin (1 g) / gentamicin (80 mg) for irrigation in 500cc of normal saline	No recurrence of capsular contracture. Haematoma (*n* = 1)
Nair^25^	5	Retrospective review; 15.34 months	N/A	Breast implant revisional surgery with capsulectomy / capsulorrhaphy	GalaFLEX (P4HB) mesh	No complication requiring reoperation. All patients reported verbal satisfaction.
Calobrace^26^	6	Retrospective review; 159 days	N/A	Popcorn capsulorrhaphy, in revisional breast augmentation	GalaFLEX (P4HB) mesh	No implant malposition or complication.
Becker^27^	21	Retrospective review; 16.5 months	Yes	Augmentation / augmentation mastopexy (*n* = 3) Mastopexy (*n* = 5) Augmentation/mastopexy revision (*n* = 13)	TIGR® Matrix surgical mesh	Asymmetry requiring revision (*n* = 2) Flap necrosis (*n* = 1) Relapse of IMF (*n* = 1)
József^28^	Mesh group: *n* = 59 Non-mesh group: *n* = 58	Retrospective review; 16 months	N/A	Mastopexy (*n* = 58), breast reduction (*n* = 17), implant and mastopexy (*n* = 42)	ULTRAPRO™ mesh	No significant differences in surgical complications. BREAST-Q questionnaire: significant differences in breast satisfaction, physical wellbeing, and sexual wellbeing (higher scores in patients in mesh group) Likert scale (3 non-involved breast surgeons): average score for mesh group 4.4, non-mesh group 3.8.
Hamdi^29^	50	Prospective observational study; 3 years	N/A	Mastopexy	Vicryl mesh in 23 patients. Mixed polyester / vicryl mesh in 27 patients.	Average self-reported score of 4.54. Assessment by blinded fellows: average score 4.32. Statistically significant differences between Vicryl and Progrip mesh (4.33 vs 4.7). Wound dehiscence in 1 patient.
Turin^30^	12 (cosmetic breast revision)	Prospective observational study; 355 days	Yes	Complex revision / Capsulorrhaphy / Implant pocket revision / Implant exchange and mastopexy	Durasorb; resorbable polydioxanone mesh	Seroma requiring aspiration (*n* = 1) None experienced recurrence of ptosis, capsular contracture, or implant malposition.
Malucci^31^	100	Prospective observational study; 14 months	Yes	Mastopexy (*n* = 79) +/- augmentation Revisional surgeries for secondary indications	GalaFLEX (P4HB) mesh	Lower pole stability: Ptosis fully corrected. Consistent maintenance of lower pole position maintained between 6 weeks and 1 year postoperative. Measurements: Differential in descent of IMF, differential in lengthening of lower pole arch.
Chiemi^32^	41	Retrospective review; 8.4 months	Yes	Primary mastopexy-augmentation	Polydioxanone (PDO) mesh	Favourable results reported in all patients Mean scar quality scaled score 4.341 (Good-Excellent)
Chiemi^33^	200	Retrospective review; 9.3 months	Yes	Breast augmentation -Smooth implants alone (*n* = 84) -Micro-textured implants (*n* = 49) -Smooth implants plus PDO internal support matrix (*n* = 67)	Polydioxanone Internal Support Matrix (PDO)	Smooth implant group with PDO mesh had the lowest scar malposition rate of 4.48 %, significant difference compared with smooth devices alone.
Abdelkader^34^	24	Prospective randomized cohort study; 36 months	N/A	Mastopexy	ADM	Suprasternal notch-to-nipple distance (median):Mesh group: increase of 1.5cm -Non-mesh group: increase of 2cm Overall complication rate: 12.5 %
Buccheri^35^	34	Retrospective review; 6–28 months	Yes	Revisional breast augmentation	GalaFLEX (P4HB) mesh	No minor or major complications. Self-reporting questionnaires show high levels of patient satisfaction.
Tomouk^36^	11	Retrospective review; 3–5 years	N/A	Revisional breast augmentation	SurgiMend ADM (*n* = 5) or GalaFLEX mesh (*n* = 6).	Two complications in the same patient. One emergency return to theatre for bleeding, one minor scar revision.
Chiemi^37^	104	Retrospective review; 8.8 months	Yes	Revision-augmentations with vertical or wise-pattern, (*n* = 74); Revision-augmentation without mastopexies (*n* = 25), revision without implant exchange (*n* = 5)	Polydioxanone (PDO) mesh	12.5 % patients required reoperation: 30.8 % due to desired size change, 69.2 % due to complications necessitating surgery. No mesh-related complications
Bistoni^38^	72	Retrospecticve review; 24.8 months	Yes	Augmentation mastopexy	GalaFLEX (P4HB) mesh	No recurrent ptosis, bottoming out, implant displacement, or capsular contracture
Harfouche^39^	89	Retrospective review; 12 months	Yes	Revisional breast augmentation	Ovine-Reinforced Hybrid Mesh (OviTexPRS); Porcine Acellular Dermal Matrix (STRATTICE)	Reduction in Baker grades reported in all patients.
Chiemi^40^	522	Retrospective review; 13.7 months	Yes	Primary breast augmentation / mastopexy-augmentation / revision-augmentations with or without mastopexy	Polydioxanone (PDO) mesh	Decreased malposition in primary augmentation; improved scarring in mastopexy.
Buccheri^41^	60	Prospective observational study; 12 months	N/A	Primary mastopexy	GalaFLEX (P4HB) mesh	Nipple-IMF distance: significant difference between mesh group and non-mesh group (unpaired): 9.57 vs 8.47 cm, at 12 months

## Results

### Study characteristics

A total of 251 articles were identified and screened using our search criteria according to preferred reporting items for systematic reviews and meta-analyses (PRISMA) guidelines (Figure 1). Thirty-one studies published from 1981 through 2025 were included (Table 1). The 31 studies included a total of 2,425 patients. There were 24 retrospective case series and 7 prospective observational studies. Three studies included a control group to establish a comparison with the mesh group.^28^^,^^34^^,^^35^ No randomised controlled study was identified in our literature search. The mean follow-up period of the included studies ranged from 159 days to 5 years. The sample size of the included studies ranged from 5 to 522.

### Surgical techniques and outcomes

Aesthetic breast surgery techniques described include implant augmentation, mastopexy, breast reduction, and revisional aesthetic breast surgeries. Various mesh materials were described in the included studies, including Marlex (Phillips Petroleum Company, Bartlesville, Okla, USA) polypropylene mesh, Polyglactine, Poly-4-Hydroxybutyrate GalaFLEX™ (Galatea Surgical, Inc., Lexington, MA, USA) mesh, vicryl mesh, Polydioxanone (PDO) mesh, TIGR® Matrix Surgical mesh (Novus Scientific Pte Ltd, Singapore), 3D Polyester, FortaPerm Biologic tissue matrix (Organogenesis, Canton, MA, USA), TYRX™ (TYRX, Inc., Monmouth Junction, NJ, USA) absorbable antibiotic-impregnated polypropylene mesh, Ovine-Reinforced Hybrid Mesh (OvitexPRS, TELA Bio, Malvern, PA, USA), and ULTRAPRO™ (Ethicon, Somerville, NJ, USA). Six studies described the use of acellular dermal matrix (ADM). Breast augmentation (with or without mastopexy) and breast implant-related revisional surgery were the most described aesthetic breast surgical techniques among the included studies, documented in 16 of the included studies. Primary mastopexy was described in 13 studies. Primary reduction mammoplasty was described in 6 studies.

### Mesh placement

In studies involving mastopexy technique, mesh was most frequently placed in the lower pole for parenchymal support.^13^^,^^14^^,^^21^^,^^22^^,^^23^^,^^28^^,^^32^^,^^34^^,^^41^ Several studies described mesh placement beneath the skin flap**,** either encircling the breast parenchyma or coned around the breast mound.^13^^,^^14^^,^^21^^,^^22^ Circumferential placement over periareolar dermal flaps has been reported by the same author for periareolar mastopexies.^12^^,^^16^ A sling configuration, with mesh anchored medially, laterally, and inferiorly, was described to provide lower pole reinforcement.^28^ Additionally, one study reported mesh fixation to medial and lateral pillars to enhance vertical projection and central breast shaping.^29^

In studies describing mesh use in primary augmentation procedures, the lower pole remained the most frequent site of mesh reinforcement, with fixation along the inframammary fold (IMF).^33^^,^^38^^,^^40^

In revisional aesthetic breast surgeries, placement of mesh often involves the lower pole^15^^,^^25^^,^^26^^,^^30^^,^^31^^,^^37^ but is also extended to the lateral, medial, or superior borders of the implant capsule, tailored to the defect.^19^^,^^20^^,^^35^^,^^36^^,^^39^^,^^40^ Chiemi et al.^40^ described the use of mesh as a “fortress” in complex revisional surgeries requiring Wise-pattern mastopexy, using two pieces of mesh both inside and outside of the pocket.

### Outcomes

All studies concluded satisfactory overall surgical outcomes (Table 1) from using mesh in aesthetic breast surgery. Subjective assessment of clinical outcome was documented in the majority of included studies (*n* = 26). Five studies recorded patient questionnaires as part of the measures of surgical outcome. No standardised outcome measure was identified among all the included studies.

*Breast augmentation (with/without mastopexy)/ implant revisional surgeries*

A total of 16 studies investigated the use of mesh in breast augmentation (with or without mastopexy) or implant-related revisional surgeries. Reported complication rates ranged from 1.6 % to 4.8 %, with documented adverse events including seroma, hematoma, asymmetry, implant malposition and capsular contracture. Despite these complications, all studies concluded that overall outcomes were satisfactory.

Chiemi et al.^32^ reported superior results with polydioxanone (PDO) mesh incorporation (*n* = 67) compared to smooth breast implants alone, with a significantly lower rate of scar malposition in the mesh group than in patients with smooth implants alone (*n* = 84). However, the same authors reported a 12.5 % reoperation rate^33^ in patients who underwent revisional breast augmentation, primarily due to either desired size change or complications necessitating surgical intervention.

Maxwell et al.^15^ conducted a retrospective review of 78 patients who underwent revisional breast augmentation and augmentation mastopexy, concluding favourable outcomes. Notably, no cases of Baker grade III or IV capsular contracture were observed at 12 months of follow-up. In a subsequent publication,^19^ the same authors analysed a larger cohort of 186 cases with a mean follow-up of 3.3 years, reporting 1.6 % incidence of Baker grade III or IV capsular contracture.

Harfouche et al.^39^ compared porcine ADM with Ovine-Reinforced Hybrid Mesh in 89 patients with Baker Grade III or IV capsular contracture who underwent revisional breast augmentation. The authors reported reduction in capsular contracture grades in all patients at 12 months of follow-up.

*Primary mastopexy*

Thirteen studies, comprising 774 patients, examined the use of mesh in primary mastopexy. Complications varied among studies. Reported events included wound dehiscence (*n* = 1),^29^ delayed healing (*n* = 1),^21^ loss of nipple sensation (up to 7.6 %), fat necrosis (up to 7.6 %),^18^ seroma (1.5 %),^12^ infection (up to 3.8 %),^18^ hematoma (1 %),^12^ and asymmetry.^27^ One study reported extrusion of mesh in two patients.^16^

Three studies^28^^,^^34^^,^^41^ included a control group (non-mesh group) for comparison, demonstrating no significant difference in surgical complications between mesh and non-mesh groups. Abdelkader et al.^34^ reported a statistically significant difference in the median increase of suprasternal notch-to-nipple distance between the mesh and non-mesh group (median 1.5 cm versus 2 cm, *p* < 0.05).

József Z et al.^28^ incorporated BREAST-Q questionnaires as part of the study outcome measures, reporting higher patient satisfaction scores in the mesh group compared with the non-mesh group. Hamdi et al.^29^ documented patient and blinded assessor satisfaction scores, demonstrating a statistically significant advantage of mixed mesh over Vicryl mesh in terms of lower pole stability and reduced elongation.

One study^32^ primarily assessed scar hypertrophy and keloid scar formation as the main surgical outcomes, concluding that PDO mesh effectively minimised poor scarring. However, no control group was included in this study.

*Reduction mammoplasty*

Among the 6 studies (170 patients) reporting outcomes in breast reduction with mesh incorporation, all concluded satisfactory outcomes. Complications recorded include seroma, haematoma, cellulitis, skin flap necrosis, mesh extrusion, and loss of areolar sensitivity. One study^11^ reported no complications. The use of mesh was reported to be associated with less frequent early ptosis and more satisfactory mammary shape.

### Conflict of interest

Among the 31 included studies in this systematic review, conflict of interest or financial association between author(s) with mesh manufacturers were reported in 18 studies, which contributes to 58 % of all included studies in this review.

## Discussion

Aesthetic breast surgeries aim to improve the aesthetic outcome of the breasts, addressing mainly volume, shape, and ptosis of breast tissue. The use of biologic or synthetic mesh has gained interest in recent decades as a scaffold to maintain breast tissue or breast implant position, thus improving the durability and stability of aesthetic breast surgery. The use of mesh has also been proposed to reduce the risk of capsular contracture related to breast implants and to reduce the incidence of ptosis recurrence. The included studies have described different types of mesh, and each has a different composition profile. There is a lack of evidence on how each type of mesh differs from each other. Biological meshes have been advertised as more biocompatible and thus cause less inflammatory response, while synthetic meshes are at risk of causing foreign body reaction,^42^ which could cause chronic inflammation and eventually lead to mesh extrusion.

Among the studies included in this systematic review, complication rates for implant augmentation procedures were generally low, ranging from 1.6 % to 4.8 %. One study^33^ reported a reoperation rate of 12.5 % in implant revisional surgeries within a follow-up period of 8.8 months. Notably, 69.2 % of those reoperations were attributed to complications. In the literature, reoperation rates for primary augmentation reach approximately 10 % within 2 years,^43^^,^^44^ while 25 % of patients may require more than one revision procedure.^45^ However, this systematic review does not allow for a comprehensive comparative analysis of reoperation rates due to limited data.

A 2019 systematic review of mastopexy outcomes reported that, in the subgroup undergoing mastopexy with glandular reshaping without mesh (*n* = 1489), the rate of wound complication was 0.7 %.^46^ Sensory changes in the nipple-areolar complex were observed in 0.9 % of cases, while fat necrosis and infection were each reported in 0.9 % and 0.1 % of patients, respectively. The highest infection rate (1.8 %) was observed in the subgroup where mesh was used. In our systematic review, studies involving mastopexy technique with mesh reported infection rates of up to 3.8 %.^18^

All the included studies in this narrative systematic review concluded with positive outcomes with the use of mesh in aesthetic breast surgery, with improved breast shape and high patient satisfaction scores. The rate of complications in all the studies was quoted as low. The use of mesh was associated with enhanced structural support, improved longevity of results and reduction in complications. However, the incorporation of mesh in aesthetic breast surgeries should be cautious as there is limited long-term data on the outcome of mesh use in aesthetic breast surgeries.

A significant limitation of this review is the predominance of retrospective case series with small sample sizes and short follow-up durations. Only three of the seven prospective studies included a control group. The majority of the included studies are of level 4 evidence. The lack of randomised controlled studies rendered it challenging to make definitive conclusions on the effectiveness of mesh use in aesthetic breast surgery.

Moreover, there is a lack of standardisation in reporting of surgical outcomes and complications among these studies. The technique of photographic documentation is not standardised among these studies, which confounds the measurements for comparison. The variety of mesh used in different studies contributed to the difficulty in analysing the effectiveness of their use in improving aesthetic breast surgery. The reported surgical outcome and complication rate associated with one type of mesh may not be applied to another type of mesh. In addition, the variation in surgical techniques described in each study and each aesthetic breast surgery is a confounding factor for this review.

It is also pertinent to note that financial interest with mesh manufacturers was reported in 18 of the 31 (58 %) included studies, significantly contributing to commercial bias. Authors of these 18 studies were either shareholders in the manufacturer companies or receivers of financial gain from the utilisation of mesh in these studies. This contributes significantly to potential effects on the overall perception of efficacy and safety of mesh use in aesthetic breast surgery.

Overall, all the included studies concluded with a favourable surgical outcome with the use of mesh in aesthetic breast surgeries. However, the outcome measure is limited by low levels of evidence, lack of standardisation of the outcome measure, lack of control group and small sample sizes.

## Conclusion and future work

This narrative systematic review analysed the safety and effectiveness of using mesh in aesthetic breast surgeries from the literature. The 31 studies included in this review, encompassing 2,425 patients, generally reported favourable outcomes. All included studies concluded that the use of mesh was associated with improved breast aesthetics and high patient satisfaction. Complications reported in these studies were infrequent, including seroma, haematoma, and infection. However, the evidence base predominantly comprises of retrospective case series and small-sample prospective studies, with significant variability in mesh types, surgical techniques, and outcome measures. The heterogeneity of study design and the lack of standardisation in outcome measures rendered it impossible to conduct a meta-analysis in this review. Additionally, a significant proportion of studies demonstrated potential commercial bias due to financial associations with mesh manufacturers. Consequently, while preliminary results are promising, the current evidence is insufficient to recommend the routine use of mesh in aesthetic breast surgery. Future research should prioritise high-quality, unbiased studies with standardised methodologies and long-term follow-up to better ascertain the benefits and risks associated with mesh in these procedures.

## Declaration of competing interest

None of the authors have any known conflict of interest.

## References

[1] Mejia Jimenez N, Patrón Gómez AS (2021). Breast aesthetic preferences: analysis of 1294 surveys. Aesthetic Plast Surg.

[2] Jr Lund HG, AL Kumpf (2010). Aesthetic breast surgery: emerging trends and technologies. Mo Med.

[3] Potter S., Conroy E.J., Cutress R.I. (2019). Short-term safety outcomes of mastectomy and immediate implant-based breast reconstruction with and without mesh (iBRA): a multicentre, prospective cohort study. Lancet Oncol.

[4] Dikmans R.E., Negenborn V.L., Bouman M.B. (2017). Two-stage implant-based breast reconstruction compared with immediate one-stage implant-based breast reconstruction augmented with an acellular dermal matrix: an open-label, phase 4, multicentre, randomised, controlled trial. Lancet Oncol.

[5] Lohmander F., Lagergren J., Roy P.G. (2019). Implant based breast reconstruction with acellular dermal matrix: safety data from an open-label, multicenter, randomized, controlled trial in the setting of breast cancer treatment. Ann Surg.

[6] Sorkin M., QiJ Kim H.M (2017). Acellulardermalmatrixinimmediateexpander/implant breast reconstruction: a multicenter assessment of risks and benefits. Plast Reconstr Surg.

[7] Clemens M.W., Kronowitz S.J. (2012). Acellular dermal matrix in irradiated tissue expander/implant-based breast reconstruction: evidence-based review. Plast Reconstr Surg.

[8] Sbitany H., Serletti J.M. (2011). Acellular dermis-assisted prosthetic breast reconstruction: a systematic and critical review of efficacy and associated morbidity. Plast Reconstr Surg.

[9] Wagner R.D., Lisiecki J.L., Chiodo M.V., Rohrich R.J. (2022). Longevity of ptosis correction in mastopexy and reduction mammaplasty: a systematic review of techniques. JPRAS Open.

[10] Whisker L., Barber M., Egbeare D. (2021). Biological and synthetic mesh assisted breast reconstruction procedures: joint guidelines from the Association of Breast Surgery and the British Association of Plastic, Reconstructive and Aesthetic Surgeons. Eur J Surg Oncol.

[11] Johnson G.W. (1981). Central core reduction mammoplasties and Marlex suspension of breast tissue. Aesthetic Plast Surg.

[12] Góes J.C. (1996). Periareolar mammaplasty: double skin technique with application of polyglactine or mixed mesh. Plast Reconstr Surg.

[13] de Bruijn H.P., Johannes S. (2008). Mastopexy with 3D preshaped mesh for long-term results: development of the internal bra system. Aesthetic Plast Surg.

[14] de Bruijn H.P., Ten Thije RHW, Johannes S. (2009). Mastopexy with mesh reinforcement: the mechanical characteristics of polyester mesh in the female breast. Plast Reconstr Surg.

[15] Maxwell G.P., Gabriel A. (2009). Use of the acellular dermal matrix in revisionary aesthetic breast surgery. Aesthet Surg J.

[16] Goes J.C., Bates D. (2010). Periareolar mastopexy with FortaPerm. Aesthetic Plast Surg.

[17] Brown R.H., Izaddoost S., Bullocks J.M. (2010). Preventing the "bottoming out" and "star-gazing" phenomena in inferior pedicle breast reduction with an acellular dermal matrix internal brassiere. Aesthetic Plast Surg.

[18] van Deventer P.V., Graewe F.R., Würinger E. (2012). Improving the longevity and results of mastopexy and breast reduction procedures: reconstructing an internal breast support system with biocompatible mesh to replace the supporting function of the ligamentous suspension. Aesthetic Plast Surg.

[19] Maxwell G.P., Gabriel A. (2013). Efficacy of acellular dermal matrices in revisionary aesthetic breast surgery: a 6-year experience. Aesthet Surg J.

[20] Pozner J.N., White J.B., Newman M.I. (2013). Use of porcine acellular dermal matrix in revisionary cosmetic breast augmentation. Aesthet Surg J.

[21] Jr Adams WP, AC Moses (2017). Use of poly-4-hydroxybutyrate mesh to optimize soft-tissue support in mastopexy: a single-site study. Plast Reconstr Surg.

[22] Jr Adams WP, R Baxter, Glicksman C., Mast B.A., Tantillo M., Van Natta B.W (2018). The use of poly-4-hydroxybutyrate (P4HB) scaffold in the ptotic breast: a multicenter clinical study. Aesthet Surg J.

[23] Pompei S., Evangelidou D., Arelli F., Ferrante G. (2018). The use of TIGR matrix in breast aesthetic and reconstructive surgery: is a resorbable synthetic mesh a viable alternative to acellular dermal matrices?. Clin Plast Surg.

[24] Ruff E.S., Hirase T., Rude M.J. (2019). Evaluation of antibiotic-impregnated mesh in preventing the recurrence of capsular contracture. Aesthet Surg J.

[25] Nair N.M., Mills D.C. (2019). Poly-4-hydroxybutyrate (P4HB) scaffold internal support: preliminary experience with direct implant opposition during complex breast revisions. Aesthet Surg J.

[26] Calobrace M.B., Mays C., Wilson R., Wermeling R. (2020). Popcorn capsulorrhaphy in revision aesthetic breast surgery. Aesthet Surg J.

[27] Becker H., Lind J.G. (2013). The use of synthetic mesh in reconstructive, revision, and cosmetic breast surgery. Aesthetic Plast Surg.

[28] József Z., Újhelyi M., Ping O. (2020). Long-term dynamic changes in cosmetic outcomes and patient satisfaction after implant-based postmastectomy breast reconstruction and contralateral mastopexy with or without an ultrapro mesh sling used for the inner bra technique. A retrospective correlational study. Cancers (Basel).

[29] Hamdi M., Chahine F., Alharami S., De Baerdemaeker R., Hendrickx B., Zeltzer A. (2021). The 10-year experience with volume distribution mastopexy: a novel, safe, and efficient method for breast rejuvenation. Plast Reconstr Surg.

[30] Turin S.Y., Gutowski K. (2022). Bioabsorbable polydioxanone mesh for soft tissue reinforcement in revisional breast surgery. Aesthet Surg J Open Forum.

[31] Mallucci P., Bistoni G. (2022). Experience and indications for the use of the P4HB scaffold (GalaFLEX) in aesthetic breast surgery: a 100-case Experience. Aesthet Surg J.

[32] Chiemi J.A., Kelishadi S.S. (2022). Never trust the skin": a rationale for using polydioxanone internal support matrix to minimize scarring in primary mastopexy-augmentation-an observational study. Aesthet Surg J Open Forum.

[33] Chiemi J.A., Kelishadi S.S. (2022). Polydioxanone internal support Matrix: a rationale for prophylactic internal bra support in breast augmentation. Aesthet Surg J Open Forum.

[34] Abdelkader R., Malahias M., Naguib I., Abdelghani S., Mastopexy Raafat S. (2022). with or without Acellular Dermal Matrix?. Plast Reconstr Surg Glob Open.

[35] Buccheri E.M., Villanucci A., Mallucci P., Bistoni G., de Vita R. (2023). Synthetic reabsorbable mesh (GalaFLEX) as soft tissue adjunct in breast augmentation revision surgery. Aesthet Surg J.

[36] Tomouk T., Georgeu G. (2023). Use of a biological scaffold in the cleavage area in complex revision breast augmentation: a surgical technique and case series. J Plast Reconstr Aesthet Surg.

[37] Chiemi J.A., Kelishadi S.S. (2023). Polydioxanone monofilament mesh: a safety net for complex breast implant revision surgery. Aesthet Surg J.

[38] Bistoni G., Sofo F., Cagli B., Buccheri E.M., Mallucci P. (2024). Artificial intelligence, genuine outcome: analysis of 72 consecutive cases of subfascial augmentation mastopexy with smooth round implants supported by P4HB scaffold. Aesthet Surg J.

[39] Harfouche C.J., Brucker M.J., Pacella S.J. (2024). A comparison of ovine-reinforced hybrid mesh (OviTex PRS) with porcine acellular dermal matrix (STRATTICE) in the treatment of advanced breast implant capsular contracture. Aesthet Surg J Open Forum.

[40] Chiemi J., Kelishadi S.S. (2024). Soft tissue support as an adjunct to implant-based cosmetic breast surgery: a 500+ case experience. Eplasty.

[41] Buccheri E.M., Lanzano G., Villanucci A., Mallucci P., Bistoni G., Hamdi M. (2025). Long-term efficacy and safety of poly-4-hydroxybutyrate (P4HB) scaffold (GalaFLEX) in mastopexy for breast ptosis: a prospective study. Aesthetic Plast Surg.

[42] Kyriakides T.R., Kim H.J., Zheng C., Harkins L., Tao W., Deschenes E. (2022). Foreign body response to synthetic polymer biomaterials and the role of adaptive immunity. Biomed Mater.

[43] Stevens W.G., Harrington J., Alizadeh K., Broadway D., Zeidler K., Godinez T.B. (2015). Eight-year follow-up data from the U.S. clinical trial for Sientra's FDA-approved round and shaped implants with high-strength cohesive silicone gel. Aesthet Surg J.

[44] Khanna J., Mosher M., Whidden P., Nguyen S., Garzon D., Bhogal M. (2019). Reoperation rate after primary augmentation with smooth, textured, high fill, cohesive, round breast implants (RANBI-I Study). Aesthet Surg J.

[45] Forster N.A., Künzi W., Giovanoli P. (2013). The reoperation cascade after breast augmentation with implants: what the patient needs to know. J Plast Reconstr Aesthet Surg.

[46] di Summa P.G., Oranges C.M., Watfa W. (2019). Systematic review of outcomes and complications in nonimplant-based mastopexy surgery. J Plast Reconstr Aesthet Surg.

